# m5C methylation modification may be an accomplice in colorectal cancer escaping from anti-tumor effects of innate immunity-type I/III interferon

**DOI:** 10.3389/fimmu.2024.1512353

**Published:** 2025-01-10

**Authors:** Yiqi Sun, Yunfei Liu, Lu Jiang, Chao Zhong

**Affiliations:** ^1^ Surgery of Traditional Chinese Medicine Department, Sichuan Provincial People’s Hospital, University of Electronic Science and Technology of China, Chengdu, China; ^2^ Department of Anesthesiology, Sichuan Provincial People’s Hospital, University of Electronic Science and Technology of China, Chengdu, China; ^3^ Traditional Chinese Medicine Department of Orthopaedic and Traumatic, Sichuan Provincial People’s Hospital, University of Electronic Science and Technology of China, Chengdu, China

**Keywords:** m5C methylation, colorectal cancer, type I/III interferon, tumor immune escape, innate immune signaling pathways

## Abstract

Colorectal cancer (CRC) is one of the most prevalent malignant tumors in the world, and its occurrence and development are closely related to the complex immune regulatory mechanisms. As the first barrier of the body’s defense, innate immunity plays a key role in tumor immune surveillance and anti-tumor response, in which type I/III interferon (IFN) is an important mediator with significant antiviral and anti-tumor functions. 5-methylcytosine (m5C) modification of RNA is a key epigenetic regulation that promotes the expression of CRC oncogenes and immune-related genes. It can enhance the proliferation, migration, and invasion of tumor cells by affecting mRNA stability, translation efficiency, and nuclear export. In addition, m5C modification modulates the activity of innate immune signaling pathways and inhibits interferon production and function, further helping tumor cells evade immune surveillance. However, there are insufficient elucidations on the interaction between m5C modification and innate immunity in CRC. In this study, the mechanism of interferon I/III in colorectal cancer was systematically reviewed and explored. This work focused on how m5C modification promotes tumor immune escape by affecting the interferon signaling pathway, thereby providing new diagnostic markers and therapeutic targets for clinical use, and enhancing the immunotherapy efficacy.

## 1 Introduction

Colorectal cancer (CRC) is one of the most common malignant tumors in the world. Its incidence rate ranks the third among all malignant tumors, and it is the second leading cause of cancer related deaths, posing a serious threat to human health and life (1). With the improvement of living standards and the change of eating habits, the incidence rate and mortality of CRC show a rapid growth trend (2). Due to the limited therapeutic effect of traditional therapies on advanced CRC, it is crucial to perform in-depth investigations on the occurrence and development mechanisms of CRC for developing new prevention and treatment methods.

One of the important functions of the immune system is to detect and remove cells that have developed oncogenic mutations. However, certain early-stage cancer cells can evade immune surveillance and develop into advanced tumors (3, 4). Innate immunity, as the body’s first line of defense against foreign pathogens, plays a key role in tumor immune surveillance and anti-tumor responses. Type I and III interferons (IFN), important mediators of innate immunity, play a key role in antiviral defense, and exert positive anti-tumor effects in suppressing tumorigenesis and progression (5–7). Type I/III interferon is an essential mediator of innate immunity. Type I/III interferons activate and regulate innate and adaptive immune responses, directly inhibit tumor cell proliferation and survival, modulate the tumor microenvironment, and enhance anti-tumor immunity through multi-level and multi-pathway molecular mechanisms (8–11). However, CRC cells often evade immune surveillance by inhibiting IFN production and function through down-regulation of IFN receptors (e.g., IFNAR1) or interfering with innate immune receptor signaling pathways (e.g., cGAS-STING, TLR, and RIG-I) (12–18). In recent years, the potential role of type III interferon (IFN-λ) in colorectal cancer has attracted much attention. IFN-λ acts mainly on epithelial cells, is tissue-specific, and can modulate mucosal immune and antiviral responses without systemic inflammation. This gives type III interferon a unique antitumor potential in barrier tissues such as the intestine (19). However, its specific mechanism of action in CRC remains to be further elucidated.

m5C modification of RNA, as an important epigenetic regulation, is important for CRC genesis, development and immune escape (20–24). Mediated by methyltransferases (such as NSUN2), m5C modification affects mRNA stability, translation efficiency, and nuclear export, promotes the expression of oncogenes and immune-related genes, and enhances the proliferation, migration, and invasion of tumor cells (25). In addition, NSUN2-mediated m5C modification modulates the activity of innate immune signaling pathways, further sustaining tumorigenesis and immunotherapy resistance (26). However, the interaction between m5C modification and innate immunity in CRC remains unclear.

In this review, the mechanism of interferon action in CRC and how m5C modification promotes tumor immune escape by affecting the interferon signaling pathway are systematically elucidated. This in-depth study of the specific mechanism of m5C modification in CRC immune escape provides new diagnostic markers and therapeutic targets for the clinic and enhances the effect of immunotherapy.

## 2 Function of type I/III interferon in CRC

Interferon (IFN) family has been widely investigated for potential therapeutic value in oncology due to its central role in the antiviral immune response. Based on the structure, receptor type, signaling pathway, and genomic location, interferons are categorized into three types: type I interferons (e.g., IFN-α, IFN-β, IFN-ϵ, IFN-κ, and IFN-ω, etc.), type II interferons (with the only member being IFN-γ), and type III interferons (e.g., IFN-λ1, IFN-λ2, IFN-λ3, IFN-λ4) (19). The signaling of both type I and type III interferons relies on different heterodimeric receptors that activate downstream immune responses through the JAK-STAT pathway, whereas type II interferons signal through different homodimeric receptors, and therefore will not be further described in this review.

### 2.1 Type I interferons and CRC

Type I interferons (IFN-α, IFN-β, etc.) are a class of potent antiviral factors generated by various cell types and play an important role especially in the early stages of viral infection (27). They activate two tyrosine kinases (i.e., JAK1 and TYK2) by binding to the cell surface receptors IFNAR1 and IFNAR2. This signaling pathway subsequently phosphorylates STAT1 and STAT2 to form the ISGF3 trimeric complex, which binds to the interferon-stimulated response element (ISRE) and initiates the expression of a series of interferon-stimulated genes (ISGs). ISGs inhibit viral replication by multiple mechanisms to inhibit viral replication, viral transcription, translation, and degradation of viral nucleic acids (19, 28).

In CRC and pancreatic cancer, downregulation of the type I interferon receptor IFNAR1 is closely associated with the accumulation of fibroblast activating protein (FAP) and extracellular matrix (ECM). IFNAR1 regulation affects the function of fibroblasts, and change matrix formation in the tumor microenvironment, thereby promoting tumor growth (29). In addition, type I interferons influence the depletion status of CD8+ T cells in chronic viral infections and cancer by regulating their terminal differentiation. Studies have shown that the phosphatase PTPN2 enhances anti-tumor immune responses by impairing type I IFN signaling and inhibiting the generation of terminally depleted CD8+ T cells (30). In CRC patients, type I interferon regulates Gzmb expression in CTLs through activation of the STAT3 signaling pathway, which in turn enhances its effector function. However, decreased IFNAR1 expression inhibits the antitumor effects of cytotoxic T lymphocytes (CTL) and evades host immune surveillance (31, 32). In addition, type I interferon prolongs the cell cycle progression of colon cancer cells, especially the prolongation of S phase and the transition to G2/M phase, through the activation of p21WAF1/CIP1. This is considered to be an important mechanism of its antitumor effects (33). Meanwhile, IFNAR signaling attenuates the function of regulatory T cells (Treg) and enhances the antiviral and antitumor activity of effector T cells in viral infections and tumor environments, leading to chronic infections and attenuation of tumor load (34). In summary, type I interferon plays a key role in the antiviral immune response, regulates the tumor microenvironment, immune cell function, and inhibits tumor progression and immune escape through multiple mechanisms.

### 2.2 Type III interferon and CRC

Similar to type I interferon, Type III interferons (IFN-λ family) activate JAK1 and Tyk2 by binding to IFNLR1 (IL-28Rα) and IL-10R2 receptors, initiating the phosphorylation of STAT1 and STAT2 to form the ISGF3 complex, which then induces interferon-stimulated gene (ISG) expression (19, 35–37). Despite the large overlap in signaling pathways between type I and type III interferons, there are obvious differences in their biological functions (19). First, type III interferon receptor expression is tissue-limited, being present mainly in epithelial cells and some myeloid leukocytes, thus focusing its action on mucosal and barrier tissues, such as the gut, respiratory tract, and skin. This allows type III interferons to play important antiviral, anti-inflammatory and immunomodulatory roles in these tissues. In contrast, the receptors for type I interferons are widely expressed in most cell types, and rapidly induce strong systemic immune responses early in viral infection. Type III interferons induce ISG at a weaker rate and intensity than type I interferons and are more inclined to maintain immune homeostasis in local tissues and to reduce systemic inflammatory responses (19, 38). This difference makes it possible for type III interferons to provide more precise immunomodulation in specific tumors, avoiding the immunopathological side effects common to type I interferons (19, 37).

With respect to colorectal cancer, researchers have verified that gamma irradiation directly induces type III interferon (predominantly IFNL1) expression in human colorectal cancer cell lines in a dose- and time-dependent manner. This process is mediated primarily by the STING-TBK1-IRF1 signaling axis (39). Type III interferon signaling further enhances IFN expression through upregulation of kinases in the STING-TBK1 signaling axis, thus creating a positive feedback loop IFN expression and forming a positive feedback loop. This mechanism provides new insights into understanding radiation-induced antitumor immunity and may offer potential applications for IFN-based cancer therapy (39). In addition, type III interferons directly induce apoptosis in cancer cells through their unique receptor complex, IFNLR1. IFN-λ signaling is pro-apoptotic by inducing cell cycle arrest and activating apoptosis-associated proteases (e.g., caspase-3, caspase-8, and caspase-9) (40, 41). Besides, PAM (an polysaccharide of atractylodes macrocephala), enhances phagocytosis of CRC cells by bone marrow-derived macrophages (BMDM) through MyD88/TLR4-dependent signaling pathway and promotes the production of pro-inflammatory factors (e.g., IL-6, IFN-λ, and TNF-α) when combined with IFN-λ in CRC. This can effectively inhibit tumor growth *in vivo* (42). Type III interferons also exert antitumor effects by down-regulating tumor-associated macrophages (TAMs), especially IL-28B (a type III interferon). IL-28B inhibits the polarization of M2-type macrophages by suppressing the STAT3 and JNK signaling pathways, thereby enhancing the antitumor immune response and delaying tumor progression (43).

In conclusion, the antitumor effects of type III interferons in colorectal cancer are reflected in the direct induction of apoptosis, enhancement of cancer cell clearance by immune cells, and suppression of the proportion of M2-type macrophages in the tumor microenvironment. These findings suggest that type III interferons have important potential in colorectal cancer treatment and may become important candidates for cancer immunotherapy.

## 3 Innate immune interferon signaling pathway and colorectal cancer

**Figure 1:**
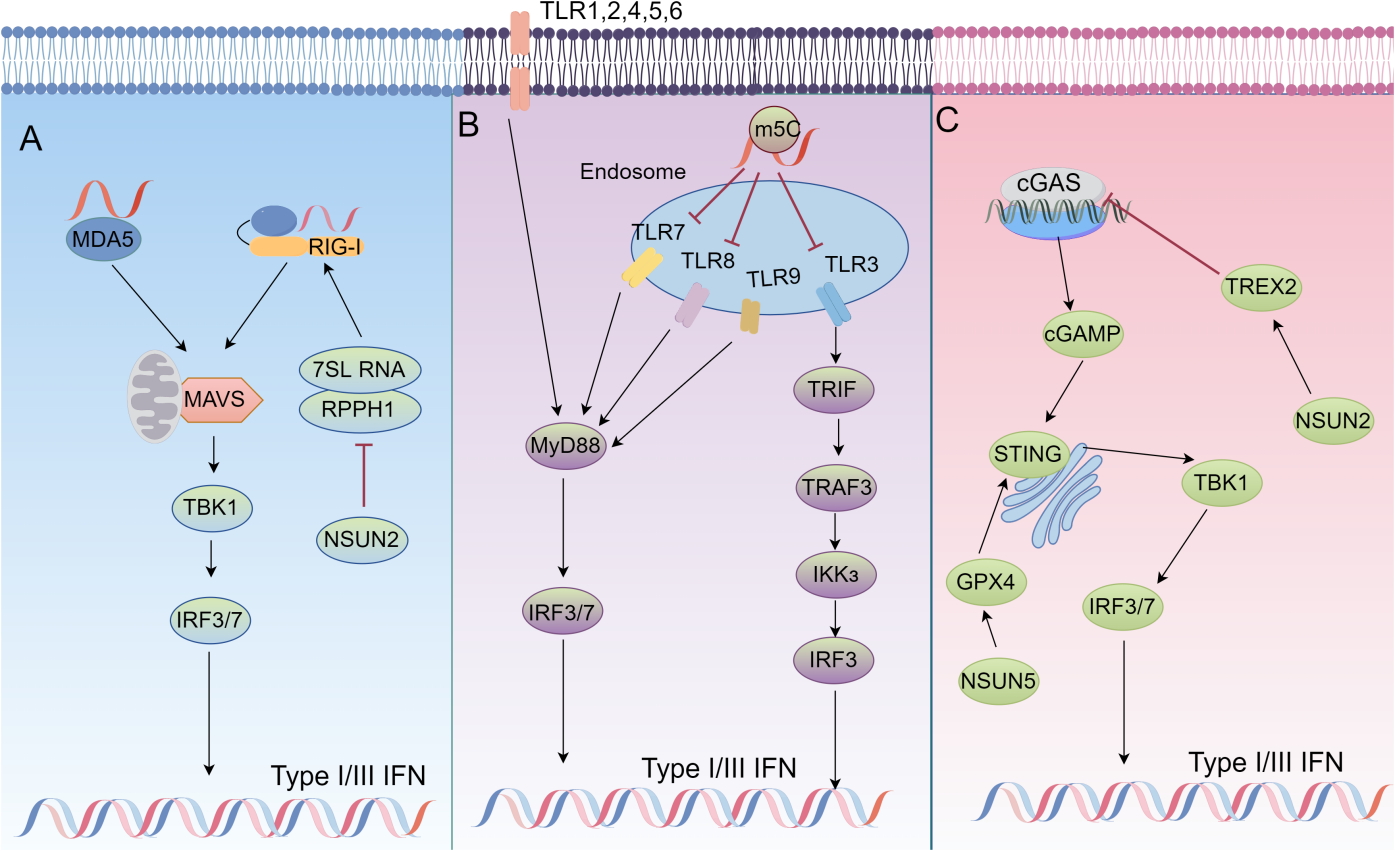
The role of m5C modifications in immune signaling regulation. **(A)** In NSUN2-deficient cells, ncRNAs transcribed by host RNA polymerase (Pol) III, particularly RPPH1 and 7SL RNA, are significantly upregulated, leading to an increase in unshielded 7SL RNA in the cytoplasm. This 7SL RNA acts as a direct ligand for RIG-I-mediated interferon (IFN) responses. In NSUN2-deficient cells, inhibiting Pol III transcription or silencing RPPH1 and 7SL RNA suppresses IFN signaling, partially rescuing viral replication and gene expression. **(B)** RNA signals through human TLR3, TLR7, and TLR8, but the incorporation of modified nucleosides such as m5C abolishes this activity. Dendritic cells (DCs) exposed to such modified RNA express significantly fewer cytokines and activation markers compared to DCs treated with unmodified RNA. **(C)** NSUN2 activation maintains global m5C RNA methylation (including TREX2) and stabilizes TREX2 to limit cytoplasmic dsDNA accumulation and cGAS/STING activation, thereby reducing interferon production and promoting tumorigenesis and resistance to anti-PD-L1 immunotherapy. Meanwhile, NSUN5-mediated 5-methylcytosine (m5C) modification of GPX4 sustains redox homeostasis in colorectal cancer (COAD) by activating the cGAS-STING signaling pathway, thereby enhancing anti-tumor immunity.

Type I/III interferon is an important component of the host’s innate immune system and plays a key role in fending off viral infections and suppressing tumorigenesis. The complex game between tumor cells and the innate immune system often determines tumorigenesis, development and progression (5, 7). It has been reported that tumor cells are able to inhibit IFN generation and the activation of the signaling pathway through interactions with the host immune system, thereby weakening the host’s immune surveillance ability. Attenuated or delayed type I/III IFN responses lead to disruption of the host immune response (44–47). This may be one of the key mechanisms by which tumor cells escape from immune surveillance. To further elucidate this mechanism, the next section focuses on the interactions between tumor cells and host innate immune signaling pathways, especially the cGAS-STING, TLR and RIG-I-like receptor (RLR) pathways (48). These pathways play a central role in the generation and regulation of IFN. Hence, exploring their roles in tumor immune escape is expected to provide new theoretical basis and therapeutic strategies for the in-depth understanding of tumor immune escape mechanisms as well as clinical treatment of tumors by IFN (**Figure 1**).

### 3.1 cGAS-STING and colorectal cancer

The cGAS-STING pathway is one of the central mechanisms by which the innate immune system recognizes cytoplasmic DNA and initiates antiviral and antitumor immune responses (49–51). Activation of this pathway is essential for cells to sense their own or foreign DNA molecules, especially in tumor cells since it plays an important role in the immune response and cell death. cGAS (cyclic guanosine-adenylate synthase) is the main receptor in the cell responsible for sensing free cytoplasmic DNA. When exogenous or endogenous double-stranded DNA (dsDNA) is present in the cytoplasm, cGAS recognizes and binds these DNA fragments, which subsequently form dimers and are aggregated by a mechanism of liquid-liquid phase separation (49). This process significantly increases the enzymatic activity of cGAS, enabling it to catalyze the cyclization of guanosine triphosphate (GTP) and adenosine triphosphate (ATP) to produce the cyclic dinucleotide cGAMP (2’3’-cGAMP). cGAMP is a second messenger capable of binding to the downstream receptor STING (interferon Gene Stimulating Factor) (52). STING is resident in the endoplasmic reticulum membrane. cGAMP binding to STING results in a conformational change that translocates it to the Golgi apparatus and recruits TBK1 (TANK-binding kinase 1) and IKKϵ (IκB kinase ϵ) (53). These kinases activate nuclear translocation of IRF3/7 by phosphorylating STING and the downstream transcription factor IRF3/7 (Interferon Regulatory Factor 3/7), which ultimately induces the expression of type I interferons and pro-inflammatory cytokines and initiates a potent antiviral and anti-tumor immune response (50, 51) (**Figure 1**).

In tumor cells, genomic instability, defective DNA damage repair, and cell cycle disorders will lead to the accumulation of large amounts of abnormal cytoplasmic dsDNA. These cytoplasmic DNAs are capable of activating the cGAS-STING pathway, which in turn initiates an immune response. This pathway acts an important role in various cancers, including colorectal, breast, and lung cancers (49–51, 54, 55). Activation of the cGAS-STING pathway has been proved to have significant potential in promoting anti-tumor immunity, especially in enhancing tumor efficacy against immune checkpoint inhibitors such as PD-1/PD-L1 antibodies (56–59). For example, in CRC, Talazoparib leads to p21 activation, stimulates inducing cellular senescence (TIS), and thus activates the cGAS-STING pathway, promotes the secretion of type I interferon (IFN-I) and stimulates anti-tumor immune responses by blocking ubiquitination of p53. Combined Palbociclib inhibition of CDK4/6 further enhanced the effect of Talazoparib, while PD-L1 antibody could enhance the activity of anti-tumor T cells by inhibiting the immune escape of tumor cells (60). Combining radiotherapy with the ATR inhibitor berzosertib activates STING signaling and enhances immunotherapy by inhibiting SHP1 function in colorectal cancer (61). Lactobacillus rhamnosus (LGG) further enhances anti-tumor immune responses, especially CD8+ T-cell activity, by activating the cGAS-STING pathway and inducing IFN-β release from dendritic cells (DCs) (58). Nanoparticle-delivered cisplatin and camptothecin can enhance antitumor immunity by inducing DNA damage and activating the cGAS-STING pathway (15). cGAS (MB21D1) and STING (TMEM173) genetic variants may affect the activation of the pathway and thus the efficacy of the immunotherapy (62, 63). RC48, as a new type of antibody-drug coupling (ADC), exhibit significant anti-tumor activity in HER2-positive colorectal cancer cells. RC48 inhibits cancer cell proliferation and enhances the sensitivity of cancer cells to immunotherapy by inhibiting the HER2-mediated cGAS-STING pathway (64). Up-regulation of BANF1 is closely related to poorer prognosis and reduced immune cell infiltration. BANF1 impedes the anti-tumor immune response by inhibiting the cGAS-STING pathway. Therefore, targeting BANF1 may be an effective strategy to enhance the efficacy of immunotherapy (59).

In summary, anti-tumor immune responses can be effectively enhanced by targeting the cGAS-STING signaling pathway. Combining these mechanisms with existing cancer therapies has important potential for clinical application and provides a theoretical basis for the design of future clinical trials.

### 3.2 TLRs and colorectal cancer

As pattern recognition receptors (PRRs), Toll-like receptors (TLRs) regulate innate and adaptive immunity by recognizing pathogen-associated molecular patterns (PAMPs), and play a key role in inflammation, infection, and the onset and progression of diverse diseases (44, 65–67). TLRs are distributed in cellular membranes (e.g., TLR1, TLR2, TLR4, TLR5, TLR6, TLR10, TLR11) or endosomal/lysosomal membranes (e.g., TLR3, TLR7, TLR8, TLR9). They can be activated by microbial components, such as lipopolysaccharides, flagellin, DNA, and RNA, as well as by some endogenous ligands (68). TLR signaling pathway is mainly activated by two major articulators, MyD88 and TRIF. MyD88 is involved in the signaling of almost all TLRs (except TLR3) and ultimately activates NF-κB, whereas TLR3 and TLR4 activate IRF3 via TRIF (69). Moreover, TLR7, TLR8, and TLR9 activate IRF7 via MyD88. These transcription factors (NF-κB, IRF3, IRF7) induce the expression of inflammatory factors, chemokines, and interferons after translocation to the nucleus (44, 66, 67) (**Figure 1**).

In the immune system, as pattern recognition receptors, TLRs recognize PAMPs and trigger innate immune responses. They are involved in anti-infection and play an important role in anti-tumor immunity (44, 66, 67). High expression of PD-L1 and TLR-4 is closely associated with poor prognosis of CRC patients. This suggests that PD-L1 can be used as an independent indicator of CRC prognosis, and PD-L1 and TLR-4 expression are closely related, providing a theoretical basis for the future use of PD-1/PD-L1 inhibitors in combination with TLR agonists (70). In recent years, TLR agonists have shown potential applications as immunostimulants in cancer therapy. L-pampo is a novel dual TLR1/2 and TLR3 agonist that induces both humoral and cellular immune responses and triggers cell death in certain cancer cells. According to RNA-seq analysis, it was found that after activating TLR signaling in immune cells, colon cancer cells and prostate cancer cells, L-pampo delivered signals through the PI3K-AKT and JAK-STAT signaling pathways in combination with reactive oxygen species (ROS) and oxidative phosphorylation (OXPHOS). Among them, this signaling pattern is particularly prominent in prostate cancer cells, suggesting that prostate cancer cells may be sensitive to L-pampo (71). TLR4 is a classical receptor for Gram-negative bacterial lipopolysaccharides (LPS), and is vital for colitis-associated cancer (CAC). TLR4 overexpression is able to promote CAC onset and progression through mechanisms such as promoting cell proliferation, inhibiting apoptosis, and accelerating invasion and metastasis (72–76). The positive feedback loop between TLR4 signaling and microRNA (miR)-155 accelerates CAC progression (77–80). In chemoresistant colon cancer cells, upregulation of TLR2/6 and TLR5 result in downregulation of miR-125b-5p and upregulation of CD248 through NF-κB activation of specific protein 1 (Sp1). This in turn enhances the drug resistance and invasive ability of cancer cells. Drug resistance of colon cancer cells can be effectively reversed by transfection of miR-125b-5p mimics or silencing of Sp1 or CD248 genes (81). Besides, the TLR7 agonist GY101, a novel selective agonist, significantly enhanced the secretion of multiple cytokines by mouse splenic lymphocytes while inhibited the growth of tumors derived from colon cancer and other less immunogenic cancer cells *in vivo* (82). GY101 has demonstrated its great potential in cancer immunotherapy by promoting lymphocyte infiltration and polarizing M2-type macrophages to M1-type macrophages (82). TLR signaling also regulates colon cancer stem cell (CSC) proliferation through the NADPH oxidase 1 (NOX1)-dependent redox epidermal growth factor receptor (EGFR) signaling pathway. NOX1 expression is restricted to proliferative CSCs and is regulated by TLR activation in response to microbiota. This reveals that the TLR-NOX1-EGFR axis serves as an important redox signaling node in CSCs for the maintenance of colonic homeostasis (83).

In summary, TLR signaling plays a key role in the activation of cancer immune response, and influences cancer development, progression and therapeutic sensitivity by regulating cancer stem cell proliferation and tumor microenvironment. In the future, the combination of TLR agonists with other immunotherapies may provide new strategies and ideas for cancer treatment.

### 3.3 RIG-I and colorectal cancer

RLR (RIG-I-like receptors) are RNA sensors in the cytoplasm and consist mainly of RIG-I, MDA5, and LGP2. RIG-I and MDA5 contain two tandem caspase activation and recruitment domain (CARD) that interact with the signaling transducer protein (84). MAVS is anchored to the mitochondrion, mitochondria associated membrane (MAM) and peroxisomes through its C-terminal transmembrane structural domains (85). In the RLR signaling pathway, RIG-I and MDA5 are activated by recognizing immunostimulatory RNAs such as viral RNAs, undergo conformational changes and multimerize their CARD structural domains (86). This in turn interacts with MAVS, transmits signals to TBK1 and IKKϵ, and ultimately activates IRF3, IRF7, and NF-κB, which collectively induce type I interferon expression (84, 86–89) (**Figure 1**).

RIG-I (retinoic acid-inducible gene I) plays multiple roles in CRC, mainly affecting tumor immune escape, inflammation and immune signaling pathways (90, 91). First, RIG-I promotes immune escape by regulating the expression of programmed death ligand 1 (PD-L1), which is an important immune checkpoint molecule for tumor cells to evade immune surveillance. Overexpression of RIG-I stabilizes PD-L1 and helps tumor cells to escape from immune clearance, yet this process does not depend on the involvement of type I interferon (IFN) signaling. In terms of the specific mechanism, RIG-I inhibits the ubiquitination and proteasomal degradation of PD-L1 by competitively binding to PD-L1 with Speckle-type POZ proteins (SPOPs), thereby maintaining its stability and ultimately promoting immune escape in CRC (17). In addition, RIG-I reduces the proliferation of CRC tumor cells by inhibiting the STAT3 signaling pathway. The DDX58 gene encoding RIG-I inhibits tumor cell growth, migration, and invasion through the STAT3/cysteine γ-cleaving enzyme (CSE) signaling pathway. This suggests that the interaction of RIG-I with STAT3 inhibits the downstream signaling pathway, thereby limiting tumor development (92). In CRC patients, frameshift mutations in the RIG-I gene lead to the formation of circular RNA (circRIG-I), which is usually strongly associated with poor prognosis. circRIG-I activates the MAVS/TRAF5/TBK1 signaling pathway by interacting with DDX3X, thereby promoting IRF3-mediated transcription of type I interferon. This process enhances the inflammatory response and accelerates the progression of CAC (93). Fascin1, as an actin-binding protein, is involved in the regulation of immune responses in CRC and regulating the migration of cancer cells. Knockdown of Fascin1 in CRC cells enhanced the RIG-I/MDA5 signaling pathway, leading to increased expression of interferon-regulated genes such as IRF-7, IFN-β, and IP-10. Fascin1 further regulates immune responses in CRC by inhibiting RIG-I signaling through interaction with IκB kinase ϵ (IKKϵ) (94). RIG-I also inhibits the killing activity of CD8+ T cells by capturing HSP90 and reduces STAT5 activation, which in turn decreases the cytotoxicity and number of tumor-infiltrating CD8+ T cells. The presence of RIG-I+ CD8+ T cells is closely associated with poor prognosis in CRC, since the activity of these cells are suppressed to make the tumor to evade clearance by the immune system (95). In DNA damage response (DDR)-associated immune activation, deletion of PARP1, a key DNA damage repair protein, triggers a p21-dependent senescence response. This in turn induces the expression of interferon-stimulated genes (ISGs) and the activation of pattern-recognition receptors (PRRs) including RIG-I. RIG-I/MAVS signaling is essential for maintaining DNA damage in the context of ISG expression is critical, reinforcing the link between DNA damage and innate immune signaling in CRC (96). PBRM1 (polybromodomain-1), a chromatin remodeling factor, represses the transcription of RIG-I and MDA5 in CRC cells. PBRM1 deficiency potentiates RIG-I-mediated innate immune signaling and enhances the inflammatory response to viral mimics. TRIM25, a ubiquitinase, interacts with and regulates the stability of PBRM1, suggesting a complex feedback loop controlling innate immune activity in CRC (97). Finally, endogenous miRNAs from the FTX locus (e.g. miR-374b and miR-545) enhance the PI3K-AKT signaling pathway associated with tumorigenesis by targeting and inhibiting RIG-I and the tumor suppressor PTEN. These miRNAs further reveal the critical role of non-coding RNAs in regulating tumor progression and the complex function of RIG-I (98). On the therapeutic side, pH-responsive nanoparticles were developed for delivering 3pRNA (RIG-I agonist) to colorectal cancer tumors. These nanoparticles can enhance cytoplasmic delivery of 3pRNA, activate RIG-I signaling, and induce immunogenic cell death in CRC. This process not only activates type I interferon and pro-inflammatory cytokines, but also enhances CD8+ T-cell infiltration and improves the efficacy of PD-1-blocking immunotherapy. Therefore, the use of nanoparticles to deliver RIG-I agonists is expected to improve the therapeutic efficacy of CRC (99, 100).

## 4 m5C Modification in CRC

### 4.1 Molecular mechanisms of m5C modification

**Figure 2:**
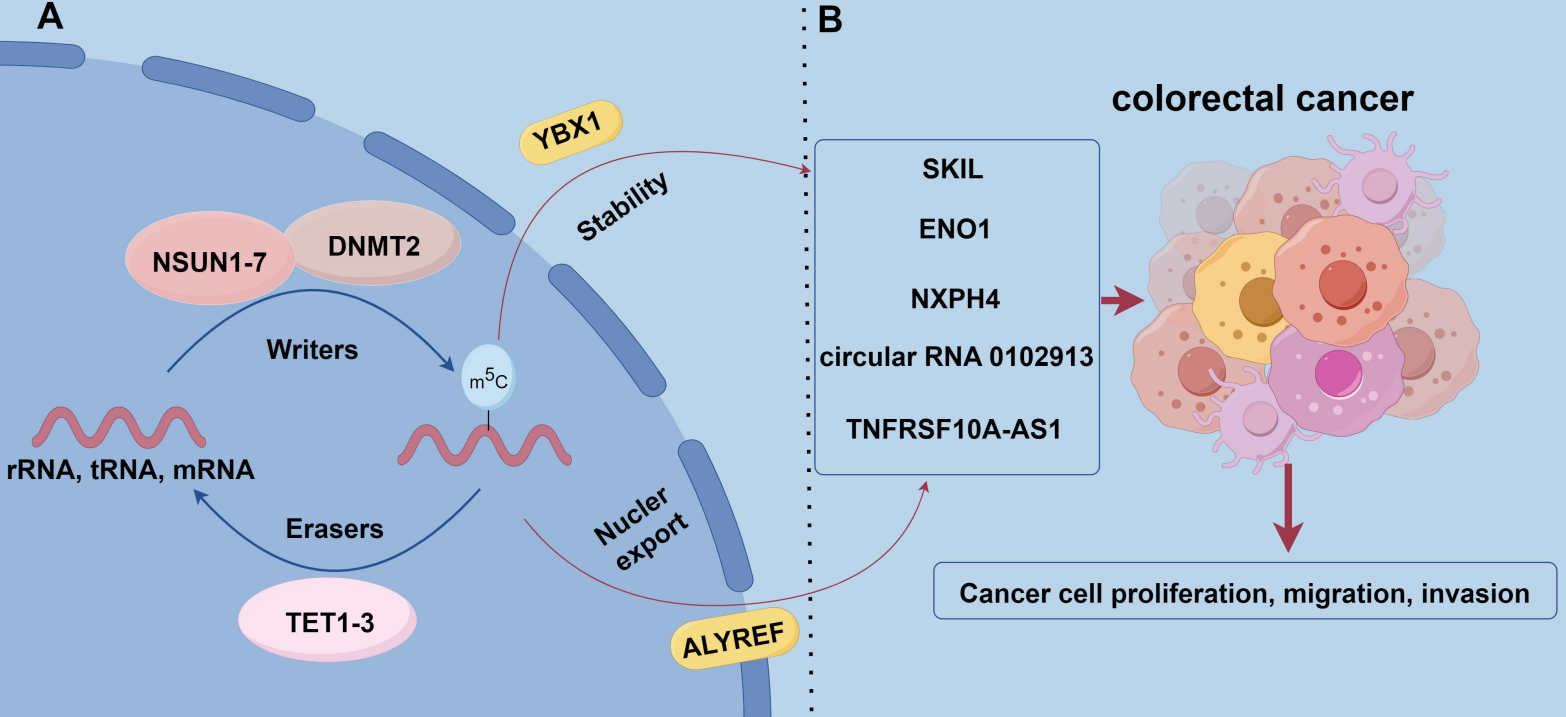
The role of m5C methylation modification in CRC. **(A)** m5C modification is catalyzed by methyltransferases that add methyl to cytosine residues of RNA using S-adenosyl-L-methionine (SAM) as a methyl donor. Known m5C methyltransferases include the NSUN family, DNMT2, and TRDMT family. allyref and YBX1 are m5C-binding proteins that regulate mRNA nuclear export as well as RNA stability and translational efficiency, respectively. the TET family (TET1, TET2, and TET3) act as m5C demethylases that remove m5C modifications through Fe (II)-dependent and α-ketoglutarate oxidation to remove m5C modifications. **(B)** NSUN2 promotes the proliferation and migration of CRC cells by modifying the mRNA of the proto-oncogene SKIL and stabilizing its expression. NSUN2 synergistically modifies the mRNA of the glycolytic enzyme ENO1 with YBX1 to enhance glycolysis and lactate production, forming a positive feedback loop that promotes tumor progression. In addition, NSUN2-mediated m5C modification increased the stability of cyclic RNA circ_0102913 and promoted the malignant behavior of CRC cells. NXPH4 is regulated by m5C modification and promotes the malignant features of colorectal cancer by inhibiting the degradation of HIF1A.TNFRSF10A-AS1 is regulated by m5C modification and affects cell proliferation and invasion, which has potential therapeutic value.

More than 170 types of chemical modifications have been identified in RNA, including N6-methyladenosine (m6A), 5-methylcytosine (m5C), and 7-methylguanosine (m7G) (101). Among them, m5C modification refers to the addition of a methyl group to the carbon atom at position 5 of the cytosine ring of RNA. This modification is reversible and dynamic, affecting the structure, stability, translocation and translation processes of RNA, and is critical for gene expression regulation and a variety of biological processes (102–105) (**Figure 2A**).

Methyltransferases (Writers): m5C modifications are catalyzed by methyltransferases, and use S-adenosyl-L-methionine (SAM) as a methyl donor to add methyl to cytosine residues of RNA (106). Currently, known RNA m5C methyltransferases include the NSUN family, the DNA methyltransferase analog DNMT2, and the tRNA-specific methyltransferase TRDMT family. Among the NSUN family members (NSUN1-NSUN7), NSUN2 is one of the most intensively studied members and can catalyze m5C modification of a wide range of RNA molecules (e.g., rRNAs, tRNAs, mRNAs, etc.). Additionally, NSUN2 exhibits a high level of expression in a wide range of tumors and is a key contributor to tumorigenesis and progression (107, 108).

Recognition proteins (Readers): m5C modification plays an important role in RNA biology, involving many cellular processes such as gene expression, RNA stability, and translational efficiency. m5C modification functions are mainly realized through binding proteins, and known m5C-binding proteins include ALYREF and YBX1. ALYREF plays an important role in the nuclear export of mRNAs, while YBX1 affects gene expression by regulating RNA stability and translation efficiency (109–111).

Demethylases (Erasers): TET family of enzymes (TET1, TET2, TET3) act as RNA m5C-modified demethylases, relying on Fe (II) and α-ketoglutarate for RNA demethylation by oxidizing m5C. TET family of enzymes plays vital roles in DNA demethylation and the process of demethylation at the RNA level. By removing m5C modifications, the TET family regulates gene expression levels and related biological functions, thereby affecting cell fate and function. This dynamic regulatory mechanism further enriches cellular responsiveness to environmental and developmental signals (112–115).

### 4.2 Molecular mechanisms of m5C RNA modification in colorectal cancer

m5C RNA modification plays a key role in the genesis, progression, and diagnosis of CRC. NSUN2 is highly expressed in CRC tissues and correlates with poor patient prognosis. NSUN2 stabilizes the expression of the proto-oncogene SKIL through m5C modification of its mRNA and promotes the proliferation and migration of CRC cells (24). NSUN2 also works with YBX1 synergistically, m5C modification of the mRNA of the glycolytic enzyme ENO1 enhances glycolysis and lactate production, forming a positive feedback loop that promotes tumor progression (116). NSUN2-mediated m5C modification increases the stability of the circRNA circ_0102913. This circRNA promotes malignant behavior of CRC cells by adsorbing miR-571 and derepressing RAC2 (117). Furthermore, m5C-mediated NXPH4 contributes to the malignant features of colorectal cancer by inhibiting HIF1A degradation (118). Long non-coding RNAs (lncRNAs) regulated by m5C modifications are also closely associated with CRC prognosis. Risk models based on m5C-associated lncRNAs can be employed to effectively predict patient prognosis and immunotherapy response (119). For instance, TNFRSF10A-AS1 is regulated by m5C modification, which affects cell proliferation and invasion and has potential therapeutic value (119). Different m5C modification patterns correlate with immune cell infiltration characteristics and patient prognosis. Patients with low m5C scores usually have more active immune responses and are more sensitive to immune checkpoint inhibitor therapy. This provides a rationale for individualized immunotherapy strategies (21). Single nucleotide polymorphisms (SNPs) may regulate mRNA levels of YBX1 by affecting m5C modification. This further affects patient response to chemotherapy and prognosis. Genetic variants in m5C modification genes may also regulate their expression and affect the CRC progression (120). m5C levels in blood immune cells of patients with CRC are significantly higher, increasing with disease progression and metastasis, and after treatment are decreased. This makes m5C a promising noninvasive diagnostic biomarker (121). The development of novel assays, such as DNAzyme-RCA-based colorimetric assays and lateral flow test strips, has enabled the convenient detection of m5C-modified miRNAs in exosomes and improved the efficiency of early CRC screening (122) (**Figure 2B**).

In conclusion, m5C RNA modification plays a key role in the biological behavior, immune regulation and therapeutic response of CRC. In-depth study of m5C modification and its regulatory factors is favorable for understanding the pathogenesis of CRC, and provides important clues for the development of new diagnostic methods and therapeutic strategies.

## 5 RNA m5C modification and interferon signaling pathway

In recent years, RNA m5C modifications have been found to play an important role in the regulation of innate immunity and interferon signaling pathways (123–125). NSUN2, an RNA m5C methyltransferase, enhances viral replication when expressed, but its deficiency leads to increased type I interferon (IFN) responses and inhibits both RNA and DNA virus replication. This effect is partly attributed to unmodified host noncoding RNAs (e.g., RPPH1, 7SL) that accumulate in NSUN2-deficient cells and directly activate RIG-I-mediated IFN signaling (126) (**Figure 1A**). During hepatitis B virus (HBV) infection, an m5C modification at nucleotide 1291 of HBV mRNA facilitates viral mRNA export and HBx translation. HBx downregulates NSUN2, reducing m5C modification of host IFN mRNA and thus decreasing IFN production, enabling sustained viral replication (127). Modified nucleosides (m5C, m6A, m5U, pseudouridine) also reduce TLR-mediated immune activation, thus limiting cytokine release and maintaining immune homeostasis (128) (**Figure 1B**). In summary, m5C modifications can modulate immune recognition of RNAs, and further balance antiviral defenses and prevent excessive immune responses.

In tumor cells, glucose acts as a cofactor for NSUN2 and binds to its amino acid 1-28 region to promote oligomerization and activation of NSUN2. Activated NSUN2 maintains global m5C RNA methylation levels and stabilizes TREX2 expression. TREX2 degrades double-stranded DNA (dsDNA) in the cytoplasm, limiting its accumulation. This inhibits the activation of the cGAS/STING signaling pathway and reduces the production of interferons. The activity of NSUN2 thus inhibits the cGAS/STING pathway and promotes tumorigenesis and progression, as well as contributing to resistance to anti-PD-L1 immunotherapy (26) (**Figure 1C**). Glutathione peroxidase 4 (GPX4) is a key antioxidant enzyme that prevents lipid peroxidation and maintains cellular redox balance. Up-regulation of GPX4 expression was found to be significantly associated with survival in colon adenocarcinoma (COAD) patients. m5C modification of GPX4 mediated by NSUN5, as well as m6A modification mediated by RBM15B and IGFBP2, facilitated activation of the cGAS-STING signaling pathway, and enhanced the anticancer immune response. m5C modification of GPX4 helps maintain cellular oxidative reduction homeostasis, activate the interferon signaling pathway, and inhibit tumor growth and spread (20) (**Table 1**; **Figure 1C**).

**Table 1:** The role of m5C methylation modification in mediating innate immune signaling pathways in disease.

m5C Modification	Related targets	Signaling pathways	Disease	Functional classification	Refs
NSUN5	cGAS/STING	GPX4	COAD	Facilitates anticancer immunity	(20)
NSUN2	cGAS/STING	TREX2	Cancer	Promote tumor progression and immunotherapy resistance	(26)
NSUN2	RIG-I	RPPH1/7SL RNA	DNA and RNA viral infection	Promote the replication and spread of the virus	(126)
NSUN2/ALYREF	RIG-I	HBx	HBV	Promote viral replication and persistent infection	(127)
m5C	TLR	TLR3/7/8	Bacteria or necrotic tissue	Inhibit the innate immune response	(128)

Therefore, an in-depth study of the relationship between RNA m5C modifications and the interferon signaling pathway is important for understanding tumor immune mechanisms. These findings provide new directions for the development of new cancer immunotherapy strategies and are expected to facilitate precision therapeutic approaches targeting RNA modifications and improve patient prognosis.

## 6 Summary and outlook

Among various RNA modifications, m6A has been extensively studied, including its role in innate interferon signaling (20, 129–135). In contrast, the direct involvement of m5C in CRC immune regulation, particularly immune escape, remains less well-documented. Nevertheless, preliminary evidence indicates that m5C modifications can influence interferon signaling pathways. For instance, NSUN2 depletion leads to the accumulation of unmodified noncoding RNAs in the cytoplasm, which act as ligands for RIG-I, thereby enhancing type I interferon responses antiviral (126). These findings suggest that m5C methylation could play a significant role in shaping interferon production and modulating immune responses.

To fully elucidate the function of m5C modifications in CRC innate immunity and tumor immune escape, more in-depth research is needed. Future studies focusing on the molecular mechanisms by which m5C modifications regulate pathways such as cGAS-STING, TLR, and RIG-I will help clarify how this modification influences the immune microenvironment in CRC. A better understanding of these mechanisms may pave the way for novel therapeutic strategies that involve adjusting m5C levels—either through targeting m5C-associated methyltransferases or demethylases, or by selectively modifying m5C sites. Such interventions have the potential to restore or enhance innate immune activity against CRC, complementing existing immunotherapies like interferon-based treatments and immune checkpoint inhibitors. As research advances, m5C-targeted approaches may contribute to more comprehensive and effective CRC treatment regimens.
